# Automatic Detection of White Matter Hyperintensities in Healthy Aging and Pathology Using Magnetic Resonance Imaging: A Review

**DOI:** 10.1007/s12021-015-9260-y

**Published:** 2015-02-04

**Authors:** Maria Eugenia Caligiuri, Paolo Perrotta, Antonio Augimeri, Federico Rocca, Aldo Quattrone, Andrea Cherubini

**Affiliations:** 1 Neuroimaging Unit, Institute of Molecular Bioimaging and Physiology, National Research Council (IBFM-CNR), Germaneto, CZ Italy; 2 Institute of Neurology, University “Magna Graecia”, Germaneto, CZ Italy

**Keywords:** White matter hyperintensities, Automated segmentation, Brain MRI, Aging, Vascular pathology, Small vessel disease

## Abstract

White matter hyperintensities (WMH) are commonly seen in the brain of healthy elderly subjects and patients with several neurological and vascular disorders. A truly reliable and fully automated method for quantitative assessment of WMH on magnetic resonance imaging (MRI) has not yet been identified. In this paper, we review and compare the large number of automated approaches proposed for segmentation of WMH in the elderly and in patients with vascular risk factors. We conclude that, in order to avoid artifacts and exclude the several sources of bias that may influence the analysis, an optimal method should comprise a careful preprocessing of the images, be based on multimodal, complementary data, take into account spatial information about the lesions and correct for false positives. All these features should not exclude computational leanness and adaptability to available data.

**Electronic supplementary material:**

The online version of this article (doi:10.1007/s12021-015-9260-y) contains supplementary material, which is available to authorized users.

## Introduction

According to the STandards for Reporting Vascular changes on nEuroimaging (STRIVE), signal abnormality of variable size in cerebral white matter (WM) that appear hyperintense on T_2_-weighted magnetic resonance imaging (MRI) without cavitation (signal different from cerebrospinal fluid) are defined as white matter hyperintensities (WMH) of presumed vascular origin (Wardlaw et al. 2013). These abnormalities have been commonly found on MRI of clinically healthy elderly people; furthermore, they have been associated with various neurological and geriatric disorders (Kim et al. 2008; Debette and Markus 2010).

MRI is highly sensitive to the changes affecting cerebral WM. Damaged white matter usually has a prolonged T_2_ relaxation time due to increased tissue water content and to degradation of the macromolecular structure of myelin. Therefore, WMH are well depicted with conventional proton density (PD) and T_2_-weighted spin echo or fast spin echo sequences, but are even more conspicuous on fluid-attenuated inversion recovery (FLAIR) images.

The presence, shape and severity of WMH might provide further insight into healthy aging and pathophysiology of various disorders. Unfortunately, since WMH patterns are heterogeneous, ranging from large confluent periventricular WMH to punctate ones in deep WM, their classification is not straightforward. It has been shown that different visual rating scales, such as the Scheltens scale or the Fazekas scale (Scheltens et al. 1998), are seldom comparable (van Straaten et al. 2006), are inappropriate when examining longitudinal progression of WMH (Prins et al. 2004), show poor sensitivity to clinical group differences (Mäntylä et al. 1997), originate high intra-subject and inter-subject variability (van den Heuvel et al. 2006) and significant ceiling/floor effects (van Straaten et al. 2006; Gao et al. 2011), therefore leading to inconsistencies between WMH studies. As an alternative to a qualitative rating scale, the manual delineation and quantification of WMH areas is a more reliable way to assess WM abnormalities, but the whole process, which is cumbersome and time-consuming for the neuroradiologist, shows high intra-rater and inter-rater variability (Grimaud et al. 1996).

For all these reasons, given the increased interest in brain research and in the context of clinical studies involving a high number of subjects, an automated approach to detection of WMH is desirable. Although several fully automated methods have been proposed for WMH segmentation, no one clearly outperformed the others. Each of the methods described in the literature has strengths and weaknesses, mainly associated with the imaging modalities used and the abnormalities detected. Several methods have been designed for lesion detection in MS patients (García-Lorenzo et al. 2013; Lladó et al. 2012), an issue that is similar to that of WMH segmentation. In practice, techniques originally trained on MS patients perform only moderately well when applied to geriatric patients (Admiraal-Behloul et al. 2005) and this happens for two main reasons. First, the contrast between gray matter (GM) and WM in MR images decreases with age. Second, the boundaries of MS lesions are sharper than those of WMH.

In this paper we describe the automated methods proposed specifically for WMH segmentation, which were applied to MR images of clinically healthy elderly people and patients with cardiovascular risk factors. Our aim is to compare the advantages and disadvantages of each algorithm, and to discuss the desirable features that should characterize an optimal detection method.

## Search Strategy and Selection Criteria

Segmentation methods for this Review were identified by searches of PubMed and Scopus between January 1980 and February 10, 2014. Since the vocabulary used to refer to WMH segmentation is extremely variable, the search terms “white matter hyperintensities” or “white matter lesions”, “segmentation” or “detection”, “automated” or “semi-automated” were used. In order to be included in our Review, methods had to be:

- Evaluated with some quantitative measure on clinical images.
- Published on a peer-reviewed journal, a book or conference proceedings (if both a journal and a conference paper existed for the same method, the journal version was cited);
- Described in an English-written paper.

Methods were excluded if published in an abstract or a thesis.

The search returned 45 studies, of which 3 were excluded because they were either abstracts or theses, and 8 because they were conference versions of a published journal article. Thus, 34 studies were selected for evaluation in this review.

## Algorithms for WMH Segmentation

The present review will be structured as follows:

- In section 3.1 the main steps of preprocessing are summarized. Since the steps that are comprised in this stage are very similar between different methods, and do not represent the true peculiarity of any segmentation algorithm, they will not be reported in detail when describing the different approaches but details can be found in supplementary Table 1;

- Section 3.2 describes the fully-automated segmentation algorithms, further sub-divided according to the learning method employed, either supervised (3.2.1) or unsupervised (3.2.2). Semi-automated algorithms, that require a certain amount of user intervention, are described in section 3.2.3;

- Section 4 gives an overview upon the evaluation metrics that are most frequently used to define accuracy (4.1) and reproducibility (4.2) of different algorithms;

- Section 5 compares the performances of automated segmentation methods applied to either elderly subjects or MS patients.

### General Preprocessing Steps

Preprocessing stages typically vary slightly across different studies, but the principal steps applied prior to the segmentation procedure are the following: registration, brain extraction, bias correction/intensity inhomogeneity correction, noise reduction and intensity normalization.

The studies considered in this review employ open source software tools for image processing well known and accepted by the MR community. All algorithms currently employed for image preprocessing generate errors, and the types and magnitudes of these errors vary within each algorithm class; moreover, errors generated by an upstream algorithm will be propagated along the preprocessing pipeline leading to additional — and often unforeseen—errors in the final output. To know which algorithms were employed and how they were "connected" in the preprocessing pipeline of the different methods, we provide a tabular representation in supplementary Table 1.

### Classification of Algorithms

Segmentation algorithms mainly rely on two broad categories of learning methods: supervised and unsupervised. Any algorithm, in turn, can be either semi-automated or fully automated, *i.e.*, requiring or not a certain amount of human intervention at some point of the processing pipeline.

Several segmentation methods are based on FLAIR images and may overestimate WMH due to their typical hyperintense appearance in cortical areas and to flow artifacts in the 4th ventricle, where a large percentage of false positives (FP) is detected. Therefore, FP correction represents an essential post-processing step when trying to segment WMH.

#### Supervised Learning Algorithms

In this paragraph we describe the principal supervised approaches for WMH segmentation; formulas defining the evaluation metrics used in each study are listed in Table 1, while values of the coefficients are reported in Table 2.

**Table 1:** Common measures used to evaluate WMH segmentation methods

Measure	Metric	Formula
Accuracy	Dice similarity index (DSC)	$\frac{2\times TP}{FP+FN+\left(2\times TP\right)}$
	Sensitivity	$\frac{TP}{TP+FN}$
	Specificity	$\frac{TN}{FP+TN}$
	Accuracy	$\frac{TP+TN}{TP+FP+TN+FN}$
	Jaccard index (JI)	$\frac{TP}{TP+FP+FN}$
Reproducibility	Coefficient of variation (CV)	$\frac{\sigma^a}{\mu^b}$

Abbreviations in formulas: *TP* number of true positives, *TN* number of true negatives, *FP* number of false positives, *FN* number of false negatives

Notes: [a] standard deviation; [b] mean

**Table 2:** Summary of results from the 34 methods described in this review, listed according to dice similarity coefficient, if known

Method	Article	Population study	Subjects	Number of subjects	Gold standard	Results	Sensitivity	Specificity	DSC^a^
Supervised	Ji et al. 2013	–	WM disease^b^	127	Manual segmentation	DSC = 0.87 ± 0.15	–	–	0.87
	Anbeek et al. 2004	–	Arterial vascular disease	20	Manual segmentation	DSC = 0.80	0.97	0.97	0.80
	Yoo et al. 2014	Korean longitudinal study on Cognitive Aging and Dementia	–	32	Manual segmentation	DSC_3T_ = 0.756 ± 0.168 DSC_1.5T_ = 0.768 ± 0.119	–	–	0.76
	Simões et al. 2013	–	HC^b^, MCI^b^	40	Manual segmentation	DSC = 0.68^c^	–	–	0.68
	Herskovits et al. 2008	ACCORD-MIND trial	Diabetes^b^	42	Manual segmentation	DSC = 0.596	-^p^	-^p^	0.60
	Dyrby et al. 2008	LADIS study	HC^b^ with WM changes	362	Manual segmentation	DSC = 0.57^c^	–	–	0.57
	Beare et al. 2009	TASCOG study	HC^b^	232	Semi-automated segmentation	DSC = 0.56	–	–	0.56
	Lao et al. 2008	ACCORD-MIND trial	Diabetes^b^	45	Manual segmentation	rho_Auto/Rater1_ = 0.79 ^d^ rho_Auto/Rater2_ = 0.74 ^d^	0.85^q^	0.99^q^	
	Maillard et al. 2008	EVA study 3C-Dijon Study	HC^b^	650 710	Visual rating	ANCOVA^e^	–	–	
	Schwarz et al. 2009	–	HC^b^, MCI^b^, Dementia^b^	114	Semi-automated segmentation	ICC = 0.916 ^f^	–	–	
Unsupervised	Jeon et al. 2010	AMPETIS study	SVD^b^	45	Manual segmentation	DSC = 0.8994 ± 0.3590	–	–	0.90
	Shi et al. 2013	-	Acute Infarction	91	Manual segmentation	DSC = 0.836 ± 0.062	0.80	–	0.84
	Khademi et al. 2012	–	Subjects with lesions	24	Manual segmentation	DSC = 0.83	0.82	0.99	0.83
	Gibson et al. 2010	–	WM disease^b^	18	Manual segmentation	DSC_FPM1_ = 0.81 ± 0.07 ^g^ DSC_FPM2_ = 0.81 ± 0.06 ^g^	–	–	0.81
	Yang et al. 2010	LEILA 75+ study	Mild/moderate dementia	30	Manual segmentation	DSC = 0.81	-^p^	-^p^	0.81
	Wang et al. 2012	Singapore aging cohort	Subjects with lesions and infarcts	272	Manual segmentation	DSC = 0.77 ± 0.06	0.81	0.97	0.77
	Admiraal-Behloul et al. 2005	PROSPER study	Risk for/pre-existing vascular disease	100	Manual segmentation	DSC = 0.75 ± 0.09	–	–	0.75
	de Boer et al. 2009	Rotterdam scan study	HC^b^	6	Manual segmentation	DSC = 0.72	0.79	–	0.72
	Samaille et al. 2012	–	MCI^b^, CADASIL^b^	67	Manual segmentation	DSC = 0.72 ± 0.16	–	–	0.72
	Seghier et al. 2008	–	HC^b^, stroke^b^, simulated data^b^	64	Manual segmentation	DSC = 0.64 ± 0.10	-^p^	-^p^	0.64
	Ong et al. 2012	–	HC^b^	19	Manual segmentation	DSC = 0.47	0.67	0.40	0.47
	Brickman et al. 2011	Clinical trial	Depression	28	Semi-automated segmentation	alpha_periventricular_ = 0.989 ^h^ alpha_deep_ = 0.981 ^h^	–	–	
	Jack et al. 2001	–	Alzheimer’s disease	19	Manual segmentation	err_overall_ =6.6 ± 9.6 % ^i^ CV = 1.4 % ^j^	–	–	
	Kruggel et al. 2008	LEILA 75+ study	HC^b^, Mild/moderate dementia	116	Manual segmentation	Se = 0.901 ^k^ Sp = 0.913 ^k^	0.90	0.91	
	Maldjian et al. 2013	Diabetes heart study	Diabetes^b^	50	Manual segmentation	rho_mean_ = 0.84 ^d^ rho_max_ = 0.87 ^d^	–	–	
	Valdés Hernández et al. 2010	–	HC^b^, stroke^b^	14	Intra-rater repeatability	SD_MCMxxxVI_ = ±326 voxel ^l^ SD_thresholding_ = ±734 voxel ^l^	–	–	
	Valdés Hernández et al. 2012	Disconnected mind project	HC^b^	20	Manual segmentation	JI_MCMxxxVI_ = 0.61 ^m^ JI_ParzenWindows_ = 0.31 ^m^	1	0.99	
	Wu et al. 2006	–	HC^b^,LLD^b^	19	Visual rating	R^2^ = 0.909	–	–	
Semi-auto	DeCarli et al. 1995	Longitudinal study of healthy aging	HC^b^	51	Operator-guided tracing	r = 0.83 ^n^	–	–	
	Kawata et al. 2010	–	SVD^b^	10	Manual segmentation	DSC = 0.772	–	–	0.77
	Itti et al. 2001	–	AIDS^b^	23	Manual segmentation	∆V = 12.8% ± 13.7% ^o^	–	–	
	Payne et al. 2002	Study of depression in later life	LLD^b^	438	Visual rating	r_Coffey_ = 0.62 ^n^ r_Boyko_ = 0.57 ^n^	–	–	
	Ramirez et al. 2011	Sunnybrook Dementia Study	HC^b^	20	Inter-rater agreement	ICC = 0.99 ^f^	–	–	
	Sheline et al. 2008	Treatment outcome in vascular depression study	HC^b^, LLD^b^	115	Manual FLAIR thresholding	*r* = 0.979 ^n^	–	–	

^a^Dice Similarity Coefficient

^b^Subjects’ clinical status. *HC* healthy controls, *MCI* mild cognitive impairment, *SVD* subcortical vascular dementia, *CADASIL* cerebral autosomal dominant arteriopathy with subcortical infarcts and leukoencephalopathy, *LLD* late-life depression

^c^Overall DSC was not available, hence the value for the last column was calculated as the average of the DSC values obtained across small/medium/large loads

^d^Spearman’s correlation coefficient

^e^Analysis of Covariance

^f^Intra-class correlation coefficient

^g^FPM1 and FPM2: False Positive Minimization methods

^h^Cronbach’s alpha

^i^Percentage of absolute error between automatic segmentation and gold standard

^j^Coefficient of variation

^k^ *Se*, *Sp* sensitivity, specificity

^l^Standard deviation

^m^Jaccard index

^n^r: Pearson’s correlation (r_Coffey_: automatic vs Coffey visual rating, r_Boyko_: automatic vs Boyko visual rating)

^o^percentage of volume difference between automatic segmentation and gold standard

^p^sensitivity and specificity values are not available, but ROC curves are provided

^q^exact values were not available, reported values are extracted from ROC curves, if the threshold was specified

One popular supervised method applied by Anbeek and colleagues (2004) used a k-nearest neighbors kNN (Duda et al. 1973) classification technique that employed multispectral information from T_1_-weighted, inversion recovery, PD, T_2_-weighted and FLAIR scans. The ground truth was defined by manual segmentation of the WMH. One fifth of the voxels were randomly selected for inclusion in the learning set, in order to reduce computation time and computer memory. Performance evaluation on the sample was made using five different feature sets, combining voxel intensities from the different sequences with spatial information (2D or 3D coordinates in either polar or Cartesian reference system) about the location of the voxel in the brain. They built a WMH probability map where the value of each voxel was defined as the fraction of hyperintense voxels among its 100 neighbors. Results showed that the best performance could be achieved using both intensity and 3D spatial features, and that the choice of the threshold for the probability maps had large influence on evaluation metrics: a higher threshold increased the specificity (less FP) at the expenses of sensitivity (more false negatives). The number of false negatives in this method was also influenced by the recruiting strategy of voxels for the training set, since the small number of samples would have unlikely been representative of real data.

Lao and colleagues (2008) built a similar classification model using a support vector machine (SVM) (Hearst et al. 1998) instead of kNN. The main difference between Anbeek’s and Lao’s approaches regarded the choice of the feature vector. In the former, neighborhood information was treated separately from the feature vector; Lao, instead, defined the feature vector by directly including intensity and spatial information about a small neighborhood of each voxel, which makes this method more robust to misregistration. A FP correction strategy was introduced using the Hilbert distance (Lin 2002).

In the context of the same population study, Herskovits and colleagues (2008) developed a Bayesian WMH-segmentation method that combined multivariate signal intensity and spatial information. In the training step, classification statistics (*i.e.*, prior probability, spatial model and signal-intensity distribution) were calculated. Afterwards, the estimated distributions were exploited to classify samples from the test set as WMH or normal tissue.

A Bayesian approach was also used by Maillard and colleagues (2008) to implement a multispectral segmentation strategy. In their method, the first step consisted in the identification of WM tissue using multispectral (T_1_, T_2_, PD) bayesian segmentation, which calculated mean and standard deviation of the distribution of each tissue class in each image modality. In a second step, WMH were identified within the WM tissue by segmenting T_2_ images, modeling two different classes of WMH voxels (low-contrast and high-contrast WMH voxels) with two gaussian distributions. Low-contrast voxels were assumed to be more probably part of a WMH’s border, while high-contrast voxels were most likely located in the core of the WMH. A second bayesian classifier was then applied to distinguish WM, low-constrast and high-contrast WMH voxels.

Dyrby and colleagues (2008) faced the segmentation issue by using an artificial neural network. The selected features included intensities of T_1_, T_2_ and FLAIR images, a 3x3 neighborhood and information about spatial location of each voxel. Six different feature vectors were composed, three including multimodal data and the other three with only FLAIR data. The authors also applied an optimal-weight-selection strategy in order to guarantee generalizability of the classifier, performed through three steps: over-fitting, pruning and selection of optimal weights. The method was then validated on a large multicenter cohort: multimodal neural networks outperformed those that were trained by FLAIR data only, and variation in MR scan quality was proven to be the largest source of error.

Simões and colleagues (2013) modeled the histogram of FLAIR intensities employing a Gaussian mixture model with three components: CSF, normal brain tissue, WMH. The traditional Expectation-Maximization algorithm was slightly modified by introducing a context-sensitive penalty term (Tang et al. 2009): this way, at each iteration of the algorithm, the probability that a voxel belongs to a certain class depends not only on the voxel’s intensity, but also on its neighbors’ current class probabilities. After convergence, they thresholded the resulting probability maps of CSF and WMH and further corrected for false positives by masking the CSF. To evaluate the performance they divided subjects according to WMH load and arbitrarily used 30 % of their dataset as a training set in order to optimize the method's parameters.

An extended FitzHugh-Nagumo reaction–diffusion model (Ebihara et al. 2003) was used by Ji and colleagues (2013) to segment WMH from FLAIR images of 127 subjects. They accounted for neighboring intensities by using an adaptive rather than fixed threshold, in order to overcome the issue of gray level intensity inhomogeneity both among different FLAIR images and within a given FLAIR. Optimized values for the adjustable parameters of the model were obtained using 30 % of the subjects as training set.

Beare and colleagues (2009) developed a method that searched for WMH per-region instead of per-voxel. They implemented a first conservative step by using a morphological watershed (Gonzalez and Woods 2002), in order to produce a segmentation with consistently defined boundaries. The features that characterized WMH reflected their topology and brightness. In the following phase, instead, they used different classifiers on the previous segmentation, in order to distinguish between real WMH voxels and FPs. They obtained the best performance using an adaptive boosting classifier (Freund et al. 1999) and a regional feature vector that comprised statistical properties of T_2_ and FLAIR intensities along with topological information, including mean WM probability in MNI space.

Differently from the main trend, Schwarz and colleagues (2009) presented a method based on run-time PD-, T_1_-, and T_2_-weighted images that could reliably detect WMH without FLAIR. The classifier learned probabilistic models of WMH spatial distribution and neighborhood dependencies from ground-truth examples of FLAIR-based WMH detections. These models were combined with a probabilistic model of the PD, T_1_, and T_2_ intensities of WMH in a Markov Random Field framework (Held et al. 1997) that allowed inference on their positions in test images. The method also performed well when training and test data were drawn from distinct scanners and subject pools.

Very recently, Yoo and colleagues (2014) developed an intensity-based, monospectral segmentation method in which the optimal intensity threshold on FLAIR images varied with WMH volume. An initial modeling of the problem was obtained by using a Bayesian decision rule on an exploratory dataset, in order to classify voxels as normal tissue or WMH. Afterwards, the search for the optimal intensity threshold was carried out on each FLAIR by testing 51 levels of threshold intensities. Validation of the algorithm was performed on two different sets of images acquired with different scanners (3 T and 1.5 T respectively). Accuracy of automated segmentation was comparable between the two validation sets.

Supervised methods have been widely used to solve automatic segmentation problems. Despite their good performances, they carry the burden represented by the need for manual segmentation, which is essential for applying any supervised learning strategy. Furthermore, these computationally intensive methods add complexity to a problem that has been shown to be solvable, with comparable accuracy and reproducibility (see Table 2), by using leaner, unsupervised methods, which we are going to describe in the following section.

#### Unsupervised Algorithms

In unsupervised segmentation, clustering techniques are generally used. This class of methods is more easily exportable to different protocols or images from different scanners (Admiraal-Behloul et al. 2005).

**Fig. 1:**
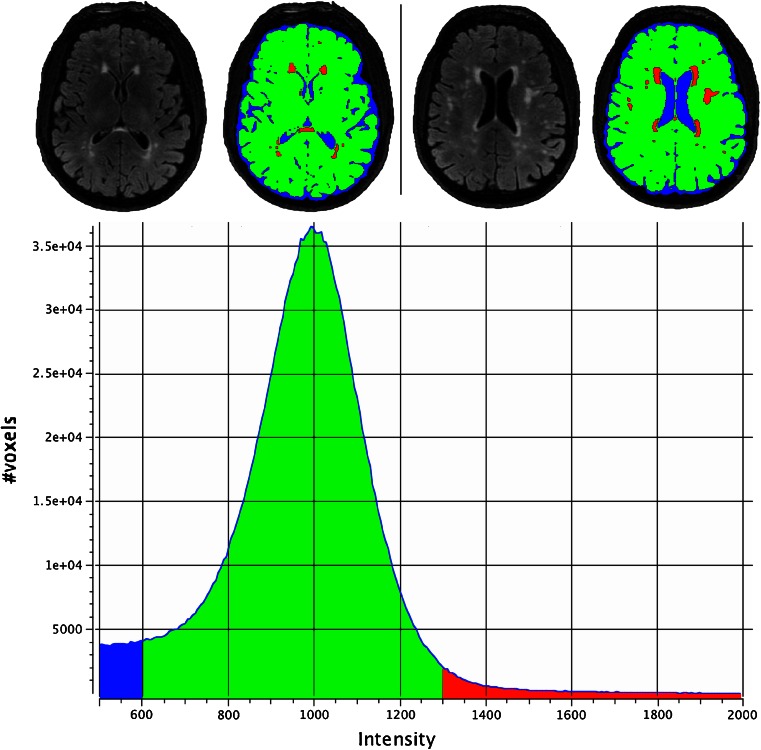
Graphical scheme of FLAIR-histoseg method. *Top panels*: two different axial slices of a FLAIR image and corresponding results of the segmentation. *Bottom panel*: histogram of the FLAIR image, intensities (arbitrary units) on the abscissa, number of voxels on the ordinate. For each FLAIR image, the thresholds used to segment the histogram in the three domains were defined by regression equations (for details see Jack et al. 2001); normal brain voxels are *green*; WMH voxels are *red*; CSF voxels are *blue*

In 2001, Jack and colleagues (2001) proposed to segment WMH by using a simple threshold derived from a regression analysis on the histogram of the FLAIR image (see Fig. 1). The descriptive parameters of the histogram were used as independent variables, while the thresholds separating CSF, normal brain tissue and WMH were the dependent variables.

A few years later, Admiraal-Behloul and colleagues (2005) developed a two-level image segmentation technique: an adaptive level that is robust to differences in image intensity ranges and image contrast, and a reasoning level that mimics expert reasoning and that remains unchanged when applied to images acquired on different MR scanners (or different software releases on the same scanner). The adaptive level mapped the exact intensity values from T_2_-weighted, PD and FLAIR images to linguistic values such as bright, dark, etc., while the reasoning level operated using these linguistic values in the fuzzy if-then rules to derive a label to every voxel. In particular, PD image intensities were used for fuzzy skull-stripping of the images, while FLAIR and T_2_ intensities were compared to differentiate WMH and CSF. The reasoning level was implemented using a well-known artificial intelligence technique: the fuzzy inference system (Mamdani and Assilian 1975; Takagi and Sugeno 1985). The number of linguistic variables, the corresponding linguistic values and the set of rules are preferably defined with the help of the expert. In this work, two experts were asked to explain how they would classify a voxel as WMH or CSF. The system was then implemented using 3 linguistic variables to classify a voxel: T_2_ intensities, FLAIR intensities and voxel position. Unlike other multispectral segmentations, these authors combined different voxel features in such a way that they were used only if crucial for the classifier: low dimensionality allows a reduction of computational time. The method also provided the possibility of setting some user-defined preferences, such as different exclusion criteria to reduce false positives.

Wu and colleagues (2006) developed an automated procedure that identified hyperintense seeds by using the intensity histogram of the FLAIR image. Seeds were labeled after thresholding with the value of the mean plus 3 standard deviations. Afterwards, they used a fuzzy connected algorithm to segment WMH while iteratively updating the seeds. When the process could no longer detect any seeds, the clusters were combined and a mask of WMH was produced.

**Fig. 2:**
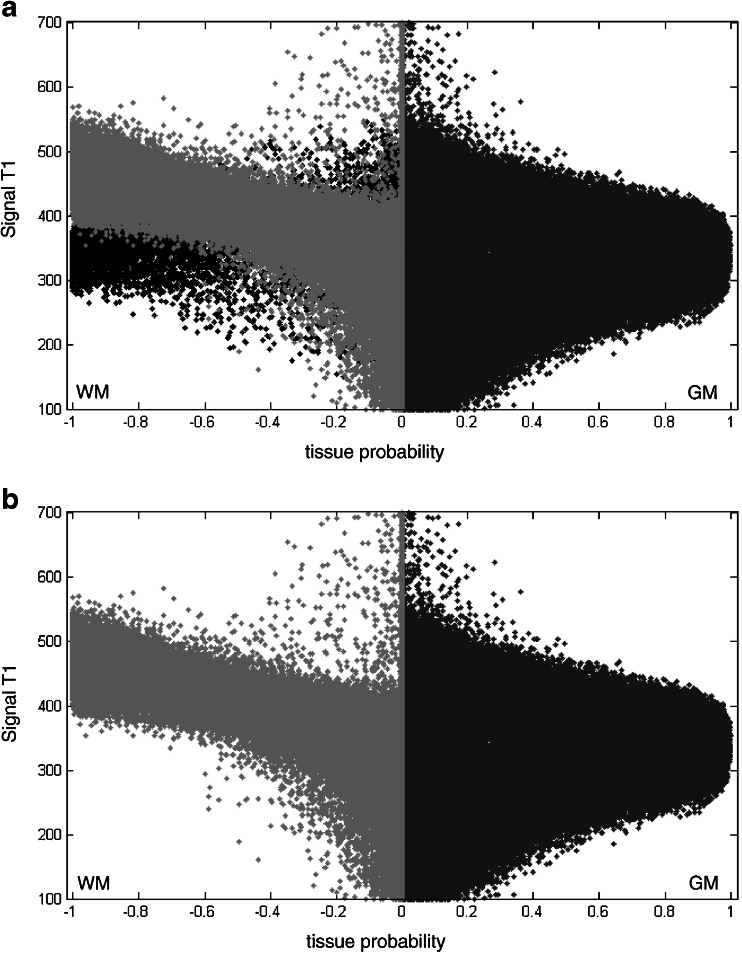
**a**: signal-to-probability maps of subject with WMH. The GM probability is shown in *dark gray* and the WM probability is shown in *light gray*. WM probability values are multiplied by −1 for display purposes. Voxels classified as WMH are shown in *black*. **b**: after removing WMH voxels, the signal-to-probability maps of the patient are comparable to those of a normal brain (both GM and WM tissues are no longer contaminated by the abnormalities). From Seghier et al. (2008)

A fuzzy classification algorithm was also used by Seghier and colleagues (2008). They exploited the normalized probability maps of GM and WM to detect outliers in each tissue class. This was obtained by comparing tissue probabilities of subjects with WMH versus WMH-free subjects under fuzzy clustering (see Fig. 2).

Another fuzzy-inference approach was developed by Gibson and colleagues (2010). Their segmentation protocol, based on FLAIR imaging, included removal of clearly hyperintense voxels, two-class fuzzy C-means clustering and thresholding to segment probable WMH. The clustering algorithm was applied twice to each voxel, in the axial and coronal planes, and the consensus of the two segmentations defined the result. This processing step increased the robustness of the final segmentation results by removing voxels incorrectly classified as hyperintense on slices with small numbers of voxels. They also tested two different FP minimization methods: both strategies involved thresholding a WM template, and the threshold for each strategy was selected by examining the results of two subjects with varying degrees of WMH burden across slices. For the first one, the segmentation results were simply masked with the thresholded template (WM probability = 0.41). For the second one, hyperintensities were removed if they were not connected in 3D to the thresholded template. As the 3D connectivity rule made the second method more liberal, a higher threshold was used (WM probability = 0.63).

Anitha and colleagues (2012) recently proposed a geostatistical fuzzy c-means clustering, obtained through the incorporation of the geostatistical estimate variance (Matheron 1963) into the objective functions of a fuzzy clustering algorithm. The proposed modification brought a decrease in FPs detection when compared to the classical fuzzy strategy.

**Fig. 3:**
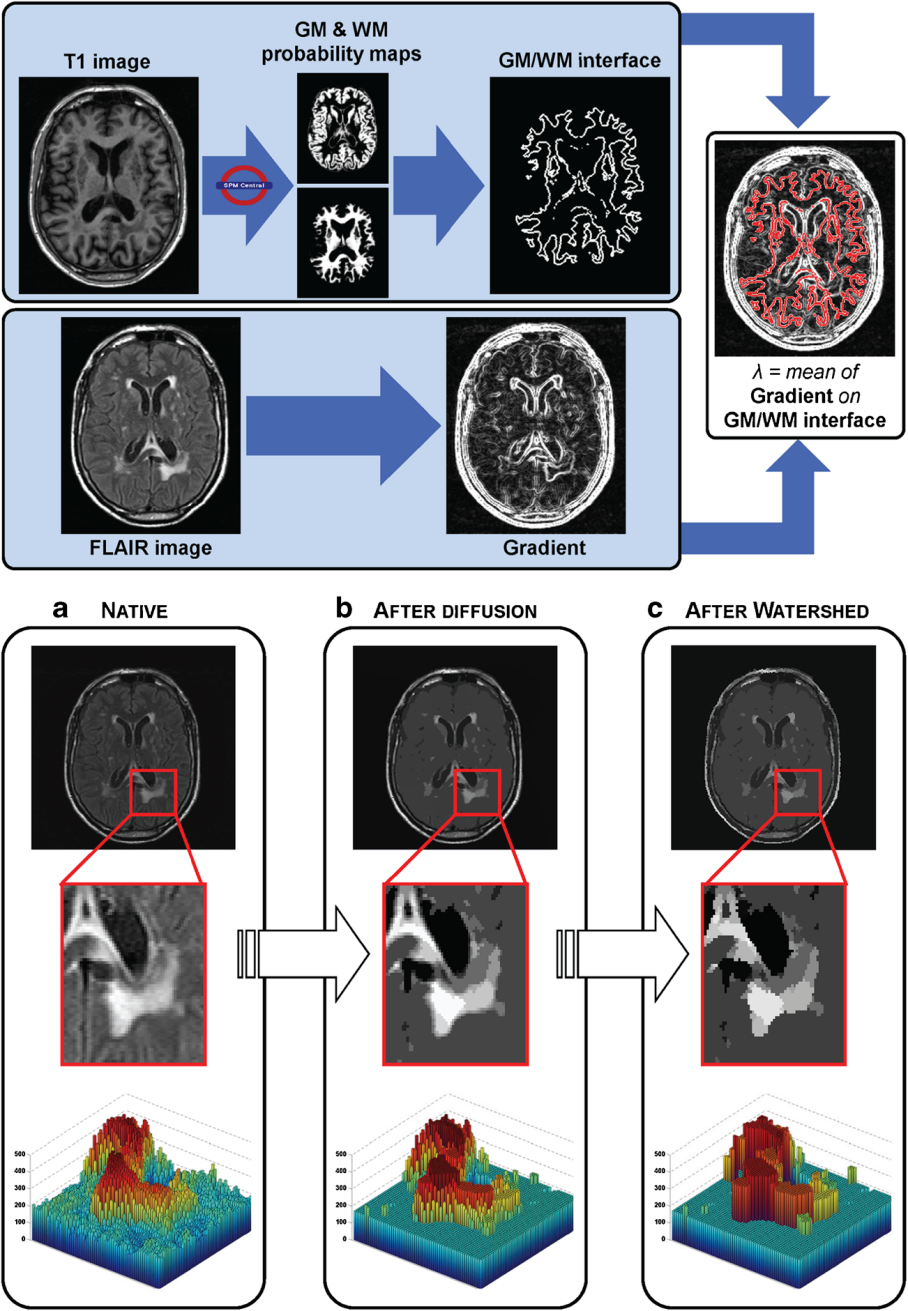
The WHASA method. *Top panel* shows the computation of the contrast parameter used for non-linear diffusion. *Bottom panel* illustrates the segmentation of the FLAIR image using non-linear diffusion and watershed. The *third row* shows a 3D visualization of the enlarged image part, where *color and height* indicate intensity values. Reproduced from Samaille et al. (2012)

Samaille and colleagues (2012) have recently developed the White matter Hyperintensities Automated Segmentation Algorithm (WHASA, see Fig. 3), specifically designed for being robust to variations due to both acquisition parameters and pathology. WHASA has been validated on a sample of 67 subjects exhibiting a broad range of WMH loads and scanned on different MRI scanners. This segmentation method exploits a non-linear diffusion framework (Perona and Malik 1990) in order to enhance contrast rather than intensity. The novelty of their method is mainly represented by the inclusion of image gradient between the features fed to the classifier, in order to include local image contrast as an important parameter. Elimination of FPs was carried out by considering the location of the WMH: segmented clusters were retained if more than 50% of their volume was located in WM.

Ong and colleagues (2012) introduced a novel method for thresholding the FLAIR intensity histogram that combined the box-whisker plot and a modified trimmed mean. They stressed that an accurate segmentation of WMH by using a thresholding technique requires the range of intensities characterizing normal brain tissue to be reliably estimated. This means that the central tendency of the voxel distribution must be determined and the trimmed mean can be used to this end. Properly truncating the ends of the histogram should allow to identify the real normal brain tissue range, hence the existence of extreme outliers of the distribution, which should be highly probable indicators of WMH.

FLAIR thresholding was also employed by Brickman and colleagues (2011) on depressed adults. First, they fitted a Gaussian curve to each hemisphere’s voxel intensity values and calculated its mean and standard deviation. Second, they defined WMH seeds as having intensity greater than or equal to 2.5 standard deviations above the mean. Finally, they combined left and right seeds and passed them individually to a region-growing algorithm that used the seed voxel intensity as its starting mean and searched for voxels that fell within 5% of this value. After these voxels were found, they were added to the image and a new mean was created. This process was iterated until all seeds had been included in the final WMH image. They classified WMH as periventricular or deep and compared results of their algorithm with those from an operator-driven quantitative approach (Gurol et al. 2006), finding a good inter-rater reliability.

Yang and colleagues (2010) also modeled WMH as outliers in the multivariate intensity distribution of healthy tissues by introducing a statistically rigorous context (Cao 1999) in which segmentation was performed. They computed the joint feature occurrence probability from T_1_ and T_2_ images. The joint intensity probability of WMH voxels was much smaller than that of healthy brain voxels, because of WMH’s small dimension and inhomogeneous intensity. The resulting probability map was modeled as a chi-squared random field; WMH were then treated as “unusual events” in this random field and a probability of being a WMH was assigned to clusters of voxels. Larger clusters of outliers were more likely to be classified as WMH.

A multistage segmentation of WMH, cortical infarcts and lacunar infarcts was recently applied to T_1_- and T_2_-weighted and FLAIR images of 272 old adults (Wang et al. 2012). The authors exploited the available software FreeSurfer (Fischl et al. 2002) to obtain the following tissue segmentations from T_1_ images: GM, CSF, WM, ventricular and subcortical structures, as well as hypointense regions. Segmentation of hyperintense regions on FLAIR images was done by applying two consecutive Gaussian mixture models, the first with three tissue classes (CSF, WM, WMH) and the second, applied only on WM and subcortical regions (identified by the previous FreeSurfer segmentation), with only two classes (normal, hyperintense). WMH and cortical infarcts are distinguished based on their location.

de Boer and colleagues (2009) extended an existing automated tissue segmentation method to WMH detection. GM, CSF and WM were segmented by an atlas-based kNN classifier on multi-modal magnetic resonance imaging data (T_1_-weighted, PD and FLAIR). This classifier was trained by non-linearly registering 12 brain atlases to the subject. The resulting GM segmentation was used to automatically find a WMH threshold on the histogram of the FLAIR scan. False positive were removed by ensuring that the hyperintensities were within WM. The method was visually validated on a set of 209 subjects.

Jeon and colleagues (2011) performed WMH segmentation by using the FAST tool of FSL (Zhang et al. 2001) on a cohort of patients with subcortical vascular dementia. The segmentation tool, based on a hidden Markov random field model, was applied to a WMH candidate region, obtained by properly refining the tissue segmentation results of a neural network. They used exclusion criteria for the subarachnoid space and brain-CSF interface of sulci to minimize FP findings. They also determined the accurate localization of WMH using an intensity-substitution method on T_1_ images.

A texture-based classification was tested by Kruggel and colleagues (2008). The distinctive texture properties of the WMH were described by means of multi-sort co-occurrence matrices (Rangayyan 2004), computed for subvolumes of the search domain (*i.e.*, WM). Each matrix yielded information about pairs of voxels in a subvolume, in particular: image intensity, gradient magnitudes and angle between gradient directions.

Another peculiar approach was developed by Valdés Hernández and colleagues (2010) and named MCMxxxVI (“multispectral coloring modulation and variance identification”). They transformed two pairs of sequences to the red-green color space, putting T_2_ through the red channel and FLAIR through the green channel so that the resulting colored volume contained the information of the fused scans. A similar process was performed for the T_1_ and T_2_ sequence pair. Since the initial fused volume had too many different levels in the red-green space, a minimum variance quantization algorithm was used to reduce color depth to a more manageable number of levels: 32 clusters provided good reproducibility of all tissue classes. Minimum variance quantization was considered an optimal clustering method because more color map entries were allocated to densely populated areas in the color space, and fewer entries were allocated to infrequent colors. At this point, clusters corresponding to WMH were selected in order to identify the maximum and minimum red-green coordinates of the tissue. The software then automatically constructed the tissue segmentation mask from the quantized volume. They also compared the performances of their method with an approach that computed multiple FLAIR thresholds for each subject and found that WMH load greatly influenced the latter method, while the MCMxxxVI was not affected. The proposed method also showed lower intra-rater variability than the thresholding technique. In a recent study (Valdés Hernández et al. 2012), this method was compared with four supervised multispectral classifiers: a back-propagated neural network, a Gaussian classifier, a kNN and a Parzen windows classifier. The MCMxxxVI performed better than the supervised techniques, but final manual editing to correct FPs was required. The best performance among supervised classifiers was achieved by the one based on Parzen windows.

Recently Maldjian and colleagues (2013) validated the Lesion Segmentation Toolbox (Schmidt et al. 2012), developed for use in the SPM8 environment, on a sample of diabetes patients. The tool calculated the FLAIR intensity distribution for each of the three tissue classes to determine outliers, weighted according to the spatial probability of being WM. This resulted in three classes of belief maps summed to generate a single belief map. A binarized version of the gray matter lesion map was used to seed a region-growing algorithm with the total belief map as target. User-selected k-thresholds were used as cutoff to generate the initial seed. The algorithm outputs WMH segmentations for each threshold, as well as a table of total WMH volume (k total volume values corresponding to each of the k thresholds). When comparing results to manual segmentation, the authors found a good correlation between the methods.

Very recently, Shi and colleagues (2013) performed WMH segmentation and infarction removal on 91 patients with acute infarction by using a coarse-to-fine mathematical morphological procedure, made of four steps: binary dilation, grayscale closing, binary reconstruction and grayscale reconstruction. After the segmentation, affected areas of acute infarctions, presented as hyperintensities on both FLAIR and diffusion-weighted images, were removed from the detected WMH.

Khademi and colleagues (2012) recently focused on computing the volume of WMH with sub-voxel precision, by accounting for the partial volume average artifact. Their method relied on an edge-based paradigm applied to FLAIR images, since the partial volume effect originates at the boundaries between different tissues. They constructed partial volume averaging maps that showed how tissue classes mix in boundary voxels, and segmented WMH assigning a value of 1 to voxels that were pure WMH, 0 to those that were not part of any WMH and an intermediate value for those that were a mixture of brain and WMH tissues.

Unsupervised methods have been successfully applied to detection of WMH, being leaner and easier to implement with respect to supervised approaches. While earlier attempts included only FLAIR intensity information (Jack et al. 2001), the importance of integrating neighborhood information has been stressed out, especially in most recent works (Anitha et al. 2012; Shi et al. 2013; Wang et al. 2012). Adding spatial information to intensity information not only makes classification methods more robust to noise (boundary detection is even more problematic in noisy images), but also takes into account the fact voxel intensities are not independent. Furthermore, a method that includes only intensity information would oversimplify the problem of WMH detection and be more prone to false positives.

#### Semi-Automated Algorithms

DeCarli and colleagues (1995) implemented a semi-automated method that exploited a double-echo pixel intensity histogram. They considered part of a WMH those pixels with intensities three or more standard deviations above the mean of the intensity-corrected histogram.

Itti and colleagues (2001) proposed a region growing algorithm that required the initial seed point to be set by an operator. Afterwards, WMH extraction was operated by flooding into neighboring voxels: the region grew from the seed point into adjacent voxels whose intensity was above an optimized threshold. The process was recursively applied until all voxels above threshold that were connected to the initial seed point had been flooded.

Payne and colleagues (2002) presented a supervised, semi-automated method for brain tissue and WMH segmentation in depressed elderly patients. Manual intervention was required both for identification of healthy tissue intensity on multimodal images and for detection of hyperintense areas on PD and T_2_ images. These candidate areas were further checked by the operator and distinguished in actual WMH and combination of tissue and CSF.

Sheline and colleagues (2008) applied a bispectral fuzzy class means based on T_1_ and T_2_ images that allowed for segmentation of the three principal tissue classes plus WMH. Identification of the centroids of each tissue class was performed by a semi-automatic peak search on the 2D histogram of T_1_ and T_2_ intensities. Their method was found to correlate well with results from simple thresholding of the FLAIR.

Ischemic WMH in subcortical vascular dementia were the focus of a study by Kawata and colleagues (2010). Initial identification of WMH candidates was performed by applying a multiple gray-level thresholding technique to an image obtained by subtracting the T_1_ image from the FLAIR. Afterwards, candidate regions were segmented with a region-growing technique from seed points detected in the previous step. After this first automated detection, a semi-automated procedure was performed to include false negatives and to remove some FPs. At this point, the authors introduced a method for adaptive selection of segmentation methods: an SVM was trained with image features extracted from each WMH region in order to select the most appropriate segmentation method for each area. There were two possible segmentation methods: a region-growing technique or a level-set method. Both methods needed initialization to be performed by a neuroradiologist.

Ramirez and colleagues (2011) developed the final component of an MRI-based processing pipeline. Their algorithm, named Lesion Explorer, was built upon two other pipeline components: an automated T_1_-based tissue segmentation protocol (Kovacevic et al. 2002); and the Semi-Automated Brain Region Extraction (SABRE) parcellation procedure (Dade et al. 2004). The segmentation was achieved by applying an adaptive local thresholding. The brain was subdivided in small 3D regions and a threshold was calculated for each of them, based on intensity histograms of PD and T_2_ images. The manual steps for checking the WMH segmentation took approximately 10–20 min of user intervention.

## Algorithm Evaluation

Two main aspects characterize the validation of a segmentation method: accuracy and reproducibility. Table 1 summarizes the principal formulas to calculate evaluation metrics, while Table 2 lists results from the studies that have been described in the previous sections.

### Accuracy

Accuracy refers to the degree of closeness of the estimated measure to the ground truth. In binary segmentation, classified samples can be true positives (TPs) and true negatives (TNs), if they have been correctly classified, or false positives (FPs) and false negatives (FNs), if there is disagreement between the gold standard and the segmentation method. In the context of WMH segmentation, TPs (WMH) are much smaller than TNs (normal appearing brain tissues), which can influence accuracy measures, especially when load is small.

In order to evaluate the accuracy of a segmentation method, WMH volume and WMH count have been tested (Goldberg-Zimring et al. 1998; Styner et al. 2008), but since they gave no information about the overlap between automatic segmentation and gold standard, the similarity index or Dice similarity coefficient (DSC) (Dice 1945) has been more widely used. The value of the index varies between 0 and 1 (perfect segmentation), with 0.7 normally considered in the literature as a good segmentation. Another frequently used measure is the intraclass correlation coefficient (ICC) (Shrout and Fleiss 1979), which is very flexible and can be adapted to different study designs (random raters, random raters from a given sample of raters, fixed raters). One caveat is that the specific design influences the way in which the metric is calculated, so it has to be carefully specified, in order for the measurement to be reliable. Unfortunately, this index is strongly influenced by the variance of the lesion load in the sample upon which it is assessed, so ICCs measured on different populations may not be comparable. Other overlap measures such as Jaccard index, sensitivity, specificity and accuracy have also been frequently used, along with the ICC and other correlation measures (see Table 1 for details).

### Reproducibility

Reproducibility represents the degree of agreement between identical experiments. In longitudinal trials, for example, physicians need to be sure that if there is any difference in WMH load between baseline and follow-up, this difference depends on physiological or pathological changes and not on the variability of the segmentation procedure. Two sources of variability are possible in the segmentation: the first is mainly due to choice of parameters in any method that requires random initialization, while the second, relative to semi-automated methods, depends on human intervention.

Reproducibility can be measured by using the coefficient of variation (CV, see Table 1) that exploits the mean and standard deviation of the different measures of total WMH load. The smaller the value of CV, the more reproducible the method is.

It is also important to note that accuracy metrics, such as DSC, may also be used for reproducibility/reliability assessment, as they depend on what data is entered into the analysis. In fact, a DSC calculated on two manual segmentations (either from the same rater at different times or from two human raters) would actually be an intra-rater or inter-rater reliability test respectively, rather than an accuracy metric.

## Application in the Segmentation of MS Lesions

Several WMH segmentation methods have been developed and applied to MS (García-Lorenzo et al. 2013; Lladó et al. 2012). Classification of methods in supervised and unsupervised ones also applies to this context. As stated in the introduction, methods that were originally designed for segmentation of MS lesions do not perform well when applied to age-related or vascular WMH. The main causes are the diminished contrast between tissue in the images of the elderly and the more fuzzy boundaries of age-related WMH.

Reported accuracies of MS methods are comparable with those summarized in Table 2. In particular, the similarity index of most MS lesion detection methods reaches or approaches 0.80, a value that was obtained in the majority of the works considered in this review, especially when analyzing subjects with medium or high WMH load. On subjects with low load, instead, all methods obtained poorer performances, probably because of the increased difficulty in detecting the fuzzy boundaries of the WMH.

Both Simoes and colleagues (2013) and Ong and colleagues (2012), by using a supervised and an unsupervised approach respectively, applied their method to the dataset used in the Medical Image Computing and Computer Aided Intervention Society’s MS Lesion Segmentation Challenge 2008 (Styner et al. 2008), consisting of 23 FLAIR images acquired at the Children’s Hospital Boston and at the University of North Carolina. The challenge contemplated the evaluation of performances through four different metrics: relative absolute volume difference, average symmetric surface distance, true positive rate and false positive rate. Results were scaled in such a way that a score of 90 points corresponded to a human rater’s segmentation. Simoes’ method scored 82.0055, while Ong’s scored 81.95, which are both very good performances. It should be noted that Ong’s method performed poorly when applied to their sample composed of 19 subjects (mean (standard deviation) age 53.15 (12.04)), among which only nine were found positive to the presence of WMH. This strengthens the notion that lesion detection in the presence of MS is less tricky than detection of age-related or vascular WMH: for this reason, methods developed for the second purpose might perform more accurately when applied to MS patients.

## Discussion

In this paper we reviewed the principal approaches to segmentation of WMH from MR images of healthy elderly subjects and patients with vascular pathologies. The need for a fast, accurate and fully automated approach has been underpinned by the efforts that are continuously made to compare and standardize the assessment of WMH load. Currently, qualitative assessment of WMH is achieved through the use of visual rating scales, although Mantyla and colleagues, by comparing 13 different scales, concluded that their heterogeneous properties resulted in inconsistencies in previous studies (Mäntylä et al. 1997), pointing out that inter-rater variability, along with time consuming procedures, made a true quantitative method superior to any visual rating scale.

Despite the number of proposed methods, an optimal algorithm has not yet been identified. This is probably due to the intrinsic complexity of the problem, as well as to the small samples upon which the majority of methods has been validated, leading to some sort of overfitting. In a recent study (Klöppel et al. 2011), supervised kNN and SVM performances (Anbeek et al. 2004; Lao et al. 2008) outperformed an unsupervised threshold approach, with SVM obtaining the best performance. It is worth pointing out that the unsupervised approach used in this study relied upon FLAIR signal intensity only, without taking into account spatial information or multispectral data. This review has presented several more sophisticated unsupervised methods that have been shown to perform as well as supervised one, without the need for a manually segmented training set.

In order to correctly validate a segmentation method, a ground truth is needed. The majority of studies exploited quantitative measures of WMH load, such as the DSC, while in others (Maillard et al. 2008; Wu et al. 2006; Kruggel et al. 2008; Valdés Hernández et al. 2010; Payne et al. 2002) the ground truth was represented by visual rating scores. For completeness, it should be noted that the DSC does not give information about over- or under-segmentation, nor provides any notion about the consistency across disease severity, as the volume correlation does. In addition, WMH in some anatomical regions are more complicated to segment than others, an issue not addressed by global measures like DSC or correlation coefficients. Hence, multiple complementary metrics are needed, and reporting the location of errors would provide a better understanding of the performance of the proposed methods.

The use of multimodal information to perform WMH segmentation has been proved to perform better than the use of a single sequence (Anbeek et al. 2004; Dyrby et al. 2008; Beare et al. 2009; Samaille et al. 2012; Valdés Hernández et al. 2010; Sheline et al. 2008), since it allows integration of spatial information, extracted from volumetric sequences, thus facilitating elimination of CSF artifacts (Anbeek et al. 2004; Dyrby et al. 2008; Gibson et al. 2010; Samaille et al. 2012; Jeon et al. 2011). Furthermore, multi-spectral segmentation also allows volume acquisition of lesion subtypes such as lacunar/subcortical infarcts and Virchow-Robin spaces (for a complete overview of lesion subtypes see Wardlaw et al. 2013). In particular, Virchow-Robin space segmentations are typically based on T_2_ and T_1_ CSF intense voxels (for a review see Valdés Hernández et al. 2013).

Redundant information is provided by PD, T_2_ and FLAIR images: in fact, WM abnormalities appear hyperintense in all three sequences. However, the extent of WMH may not look the same on the different sequences (Filippi et al. 1996a, b), and it was shown that FLAIR imaging present some limitations: it is less sensitive in the posterior fossa (Gawne-Cain et al. 1998) and for thalamic lesions (Bastos Leite et al. 2004), may overestimate WMH load and has a higher inter-vendor variability (Filippi et al. 1996a, b, 1999; Bastianello et al. 1997; Rovaris et al. 1999). Furthermore, FLAIR may present hyperintense artifacts that can lead to an increase in false positives. This alternating behavior of FLAIR, *i.e.*, losing sensitivity or specificity according to the spatial location of the lesion, might have a biological, albeit still unclear, explanation. A recent study (Haller et al. 2013) hypothesized that WMH identified by FLAIR despite the relatively mild demyelination, shown by histopathological comparison, could depend on a relatively high concentration of interstitial water in periventricular/perivascular regions, due, in turn, to age-related modifications in blood–brain-barrier and plasma properties. On the other hand, the lack of sensitivity in the thalamus and in infratentorial regions could reflect different relaxation characteristics of both normal-appearing and abnormal tissue, accompanied by age-related changes in relaxation times (Bastos Leite et al. 2004). Finally, during the acquisition of the FLAIR sequence, even a slight spatial inhomogeneity of the inversion pulse profile could influence the sensitivity of FLAIR to WMH in different spatial locations. For all these reasons, the combination of FLAIR with a redundant source (*i.e.*, T_2_) will increase the certainty of the WMH delineation and reduce false positives.

The accuracy of an automated WMH segmentation procedure is also limited by the scarce knowledge about the spatial distribution of the hyperintensities. Although it is well known that certain locations are more relevant than others (*i.e.*, periventricular watershed), the exact probability of finding a WMH in a specific region is unknown. At the moment, the automatic spatial subdivision of WMH according to their distance from the ventricles has been developed in several methods (Anbeek et al. 2004; Maillard et al. 2008; Brickman et al. 2011; Jeon et al. 2011; Payne et al. 2002; Ramirez et al. 2011), but further exploration of WMH topographic distribution is required.

In summary, whether supervised or not, from a methodological point of view a good segmentation method should:

- Rely on a good pre-processing stage, in order to be robust to noise;

- Be computationally lean;

- Use multimodal, complementary data. Anyway, since multimodal images are not always acquired in clinical practice, a good method should be adaptable to the data available in the specific context;

- Consider spatial information, both in terms of tissue classes and known topology of WMH;

- Be reproducible and applicable to data acquired at different times from different scanners;

- Rely on a good, automated method for removing false positives.

In conclusion, advances in new algorithms, as well as new developments in MRI acquisition protocols, should help neuroradiologists to improve the evaluation of WMH, both in clinical studies, investigating their relationship with normal aging and pathology, and in every-day clinical practice.

## Electronic supplementary material

Below is the link to the electronic supplementary material.

Supplementary Table 1 For each study included in the review, we list the different algorithms used for each step of the preprocessing stage. (DOC 77 kb)
