# Supplementary material for: Automatic Detection of White Matter Hyperintensities in Healthy Aging and Pathology Using Magnetic Resonance Imaging: A Review
Source: Neuroinformatics. 2015 Feb 4;13(3):261–76. doi: 10.1007/s12021-015-9260-y (PMC4468799; doi:10.1007/s12021-015-9260-y)
Supplement: Supplementary file 1 — For each study included in the review, we list the different algorithms used for each step of the preprocessing stage. (DOC 77 kb) [file 12021_2015_9260_MOESM1_ESM.doc]

Supplementary table 1: For each study included in the review, we list the different algorithms used for each step of the preprocessing stage.

| Study cited | Algorithm used for | | | | |
| --- | --- | --- | --- | --- | --- |
|  | Registration | Brain Extraction | Bias Correction | Noise Reduction | Intensity Normalization |
| Ji et al. 2013 | - | FSL-BET | - | Anisotropic Diffusion Equation | Extended FitzHugh-Nagumo Equations (step 1) |
| Anbeek et al. 2004 | Rigid registration | Mbrase | - | - | Histogram matching |
| Yoo et al. 2014 | SPM8 | SPM8 (*spm_run_preproc*) | SPM8 (*spm_run_preproc* optional) | - | - |
| Simoes et al. 2013 | - | FSL-BET | FSL-FAST | - | - |
| Herskovits et al. 2008 | Maximization of mutual information | FSL-BET | - | - | Histogram matching |
| Dyrby et al. 2008 | SPM2 | SPM2 | N3 | - | z-score standardization |
| Beare et al. 2009 | SPM5 | SMP5 | Parametric Bias Field Correction (PABIC) | - | Median and interquartile range normalization |
| Lao et al. 2008 | FSL-FLIRT | FSL-BET | N3 | Gaussian smoothing | Histogram matching |
| Maillard et al. 2008 | AIR 2.0 + MULTI PURPOSE MATCH | ATOMIA (in-house) | Bias modeling with third-degree polynomial (in-house) | - | - |
| Schwarz et al. 2009 | Maximization of mutual information / Nonlinear alignment to minimum deformation template | Manual | - | - | - |
| Jeon et al. 2010 | Rigid and affine linear transform | FSL-BET | N3 | - | Not specified |
| Shi et al. 2013 | Maximization of mutual information | FSL-BET | FSL-FAST | - | Not specified |
| Khademi et al. 2012 | - | FSL-BET | - | Bilateral filtering | - |
| Gibson et al. 2010 | - | FSL-BET | FSL-FAST + N3 | Edge- preserving anisotropic diffusion filtering | - |
| Yang et al. 2010 | Maximization of mutual information | FSL-BET | N3 | - | - |
| Wang et al. 2012 | FSL-FLIRT | FSL-BET | N3 | - | - |
| Admiraal-Behloul et al. 2005 | Rigid-body (AIR) | Fuzzy clustering | - | - | - |
| de Boer et al. 2009 | Rigid-body | Manual + Elastix | N3 | - | Range matching |
| Samaille et al. 2012 | SMP8 *coreg* function | SPM8 | SMP8 New Segment Module | - | - |
| Seghier et al. 2008 | - | SPM5 | SPM5 | - | - |
| Ong et al. 2012 | - | Model-based level set (MLS) | N3 | - | - |
| Brickman et al. 2011 | FSL-FLIRT | FSL-BET | - | - | - |
| Jack et al. 2001 | - | Manual | Mean filtering | Edge-preserving anisotropic filtering | - |
| Kruggel et al. 2008 | 9-dof transform | - | Fuzzy segmentation | - | Histogram matching |
| Maldjian et al. 2013 | SPM8 Lesion Segmentation Toolbox | - | SPM8 New Segment Module | - | - |
| Valdes-Hernandez et al. 2010 | Analyze 8.1 3D Surface Registration module | Analyze 8.1 Object Extraction Tool | - | - | - |
| Valdes-Hernandez et al. 2012 | FSL-FLIRT | Analyze 8.1 Object Extraction Tool | - | - | - |
| Wu et al. 2006 | FSL-FLIRT + ITK | FSL-BET + ITK | - | - | - |
| DeCarli et al. 1995 | - | Manual | Filtering | - | - |
| Kawata et al. 2010 | - | Histogram thresholding | - | - | - |
| Itti et al. 2001 | - | - | - | - | - |
| Payne et al. 2002 | - | - | - | - | - |
| Ramirez et al. 2011 | Rigid-body transform | BrainSizer | Gaussian fitting | - | - |
| Sheline et al. 2008 | Affine transform | Fuzzy class means | Parametric bias field correction | - | - |

- : description of this step was not available in the corresponding paper.

“not specified”: the step was included in the pipeline but there is no specification about the algorithm used.

Abbreviations: BET = Brain Extraction Tool; Mbrase = Automatic Morphology-based Brain Segmentation; SPM = Statistical Parametric Mapping; FMRIB = Functional MRI of the Brain; FAST = FMRIB's Automated Segmentation Tool; N3 = Non-parametric Non-uniform intensity Normalisation; FLIRT = FMRIB's Linear Image Registration Tool; AIR = Automated Image Registration; ITK = Insight Segmentation and Registration Toolkit.
